# *Tropheryma whipplei* in Fecal Samples from Children, Senegal

**DOI:** 10.3201/eid1506.090182

**Published:** 2009-06

**Authors:** Florence Fenollar, Jean-François Trape, Hubert Bassene, Cheikh Sokhna, Didier Raoult

**Affiliations:** Université de la Méditerranée, Marseille, France (F. Fenollar, J.-F. Trape, H. Bassene, C. Sokhna, D. Raoult); Pôle de Maladies Infectieuses, Marseille (F. Fenollar, D. Raoult); Institut de Recherche pour le Développement, Dakar, Senegal (J.-F. Trape, H. Bassene, C. Sokhna, D. Raoult)

**Keywords:** Enteric diseases, bacteria, Tropheryma whipplei, gastroenteritis, diarrhea, children, Senegal, expedited, dispatch

## Abstract

We tested fecal samples from 150 healthy children 2–10 years of age who lived in rural Senegal and found the prevalence of *Tropheryma* *whipplei* was 44%. Unique genotypes were associated with this bacterium. Our findings suggest that *T. whipplei* is emerging as a highly prevalent pathogen in sub-Saharan Africa.

*Tropheryma whipplei* is known mainly as the bacterial pathogen responsible for Whipple disease (*1*). Until recently, it was thought to be a rare bacterium typically causing disease in white men (*1*). However, recent studies have shown 1%–11% prevalence of the bacterium in fecal samples from the healthy general adult population in Europe (*2*,*3*). *T. whipplei* also was viable in a fecal sample from a patient with Whipple disease (*4*). In addition, *T. whipplei* DNA has been detected in sewage and is more prevalent in fecal samples of sewer workers (12%–26%) than in the general population, supporting this environment as a likely ecologic niche (*2*,*3*). *T. whipplei* was highly prevalent in fecal samples of children 2–4 years of age in France who have gastroenteritis but was not detected in a control group of children of the same age who did not have diarrhea (*5*).

For these reasons, we speculated that if *T. whipplei* is transmitted through the fecal–oral route, it might be more prevalent in countries with poor sanitation, such as developing countries. Because no information is available about *T. whipplei* and Whipple disease in Africa, we conducted a study to assess the prevalence of *T. whipplei* in fecal samples from healthy children in Senegal, specifically in the villages of Dielmo and Ndiop (Figure 1).

**Figure 1:**
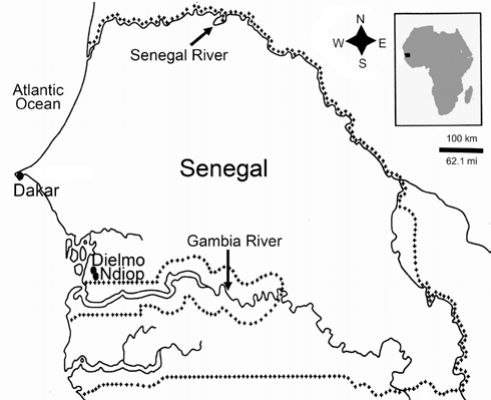
Location of Dielmo and Ndiop in Senegal, Africa. Plus-symbol lines define the Senegal frontiers. The number of fecal samples positive for *Tropheryma whipplei* and number tested for children in each age group was as follows: Dielmo, 1/13 from children <8 months of age, 5/9 from children 8–24 months of age, and 19/54 from children 2–10 years; Ndiop, 1/5 for children <8 months of age, 5/18 from children 8–24 months of age, and 27/51 from children 2–10 years of age.

## The Study

In early April 2008, we sampled fecal specimens from 150 healthy children (79 girls) 2 months–10 years of age (mean 3.5 years ± 2.5 years) living in 2 villages in Senegal (Ndiop, 77 children; Dielmo, 73 children) (*6*). These villages are included in the Dielmo project, initiated in 1990 for long-term investigations of host–parasite associations in the entire village population, which was enrolled in a longitudinal prospective study (*6**,**7*). At the beginning of the current study, parents or legal guardians of all children gave individual informed consent. The national ethics committee of Senegal approved the project (*6*). Eight wells in the 2 villages (5 from Dielmo, 3 from Ndiop), which are the only sources of drinking water for the communities, also were sampled.

After collection, each fecal specimen was mixed with 2.5 mL of absolute ethanol for storage and transportation to our laboratory at room temperature. On arrival, DNA was extracted by using the BioRobot MDx workstation (QIAGEN, Valencia, CA, USA) in accordance with the manufacturer’s recommendations and protocols. *T. whipplei* quantitative PCR assays were performed as previously described (*8*). A case was defined as 2 positive quantitative PCR results in assays targeting 2 different *T. whipplei* DNA sequences. *T. whipplei* genotyping using 4 variable sequences was performed by using samples from children with high bacterial loads, as previously reported (*9*).

Among the 150 healthy children, the prevalence of *T. whipplei* was 11% (2/18) in children <8 months of age, 37% (10/27) in children 8–24 months of age, and 44% (46/105) in children 2–10 years of age (Figure 1). None of the 8 water wells sampled were positive for *T. whipplei*.

**Figure 2:**
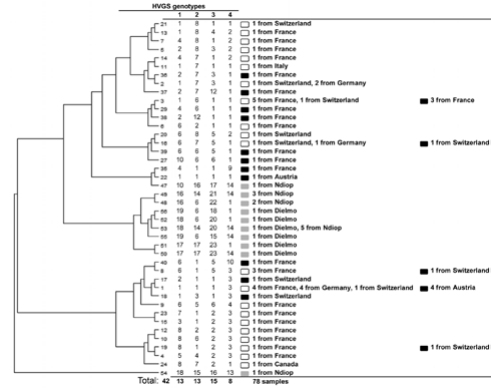
Dendrogram constructed using the unweighted pair group method with arithmetic mean showing the phylogenetic diversity of 42 genotypes from the 78 *Tropheryma whipplei* strains detected in 18 healthy children (gray squares) in Senegal, 39 adults in whom Whipple disease was diagnosed (white squares), and 21 symptomatic carrier adults (including 11 sewer workers; black squares) from Europe. Phylogeny assembly was based on the sequences of 4 variable sequences, which were concatenated to construct the dendrogram.

A complete genotype was obtained from 18 healthy children (Figure 2). We found 10 new genotypes in 3 independent clusters, all of which were unique to Senegal. Four genotypes were detected only in Ndiop and 5 only in Dielmo. One genotype common to both villages was classified as epidemic to Ndiop.

## Conclusions

In our study, 44% of children >2 years of age who live in rural Senegal carried *T. whipplei*. Our requirement for 2 positive test results ruled out sample contamination, and the 4 different PCR amplifications and sequencing reactions used for genotyping resulted in the discovery of novel sequences. This pathogen in Europe, Asia, and America has been reported at much lower prevalences than we found in Senegal (*1*–*3*,*5*). The unique genotypes we discovered in Senegal have not been demonstrated elsewhere through global DNA-based comparisons. This specific type of geographic distribution of genotypes also has been reported for *Mycobacterium tuberculosis,* for which researchers named genotypes using the same genotyping method (*10*). Clonal diffusion of a specific genotype within a single area favors a human-to-human transmission hypothesis, which the circulating clones exemplified in Senegal.

In Senegal, children are contaminated with *T. whipplei* at an early age, and the high carriage rate we observed indicates a potential public health problem. *T. whipplei* may be responsible for numerous undiagnosed infections, including gastroenteritis, in Africa. The classic form of Whipple disease, characterized by histologic periodic acid-Schiff–stained bacilli in infected small-bowel macrophages, may represent only 1 rare clinical variant of infection that *T. whipplei* can cause. The higher number of Whipple disease cases in white men may be related to a genetic factor or might reflect a large number of unrecognized cases in developing countries. However, *T. whipplei* also can cause localized infections, such as endocarditis, spondylodiscitis, meningoencephalitis, and uveitis (*1*). The bacterium also was detected in a child with pneumonia who resided in the United States (*11*).

Our study provides evidence that *T. whipplei* is common in fecal samples from children in Senegal and that local strains circulate in the 2 villages investigated. We suspect *T. whipplei* infection results directly from human-to-human transmission because water from all 8 village wells tested negative by PCR. *T. whipplei* is present throughout the world, and specific genotypes often are linked to geographic sources. We speculate that *T. whipplei* infection might be a major public health concern in sub-Saharan Africa. Additional studies are needed to investigate the role of this extremely common emerging pathogen in developing countries.
